# An inhibitor of programmed death ligand 1 enhances natural killer cell-mediated immunity against malignant melanoma cells

**DOI:** 10.1371/journal.pone.0248870

**Published:** 2021-04-01

**Authors:** Young Shin Lee, Woong Heo, Ho-Jung Choi, Hae-Ryung Cho, Ji Ho Nam, Yong Gan Ki, Hong-Rae Lee, Woo-Chang Son, You-Soo Park, Chi-Dug Kang, Jaeho Bae

**Affiliations:** 1 Department of Biochemistry, Pusan National University School of Medicine, Yangsan, Republic of Korea; 2 Department of Molecular Cell Biology and Genetics, Plus Biomedical Science Education Center, Pusan National University School of Medicine, Yangsan, Republic of Korea; 3 Department of Radiation Oncology, Pusan National University School of Medicine, Yangsan, Republic of Korea; 4 Department of Research Center, Dongnam Institute of Radiological and Medical Sciences, Busan, Republic of Korea; Duke University School of Medicine, UNITED STATES

## Abstract

Since ionizing radiation has showed the dramatic effect to kill the cancer cells through direct DNA damage as well as triggering anti-cancer immune responses including induction of NKG2D ligands, it has used for long time to treat many cancer patients. However, it has been known that radiotherapy might promote the remnant cancer cells to escape immune system and metastasis. One of the suggested ways of immune evasion is induction of a ligand for programmed death-1 (PD-L1) in head and neck cancer, bladder cancer and lung cancer cells which engages the receptor, programmed death-1 (PD-1) in immune cells. PD-1/PD-L1 axis transduces the inhibitory signal and suppresses the adaptive immunity. However, their role in innate immunity remains poorly understood. Therefore, we investigated whether ionizing radiation could change the expression of PD-L1 in malignant melanoma cells and the receptor, programmed death-1 (PD-1), in NK-92 cells. Surface PD-L1 levels on melanoma cells were increased by ionizing radiation in a dose-independent manner but the level of PD-L1 was not changed significantly in NK-92 cells. Radiation-induced PD-L1 suppressed the activity of the NK-92 cells against melanoma cells despite of upregulation of NKG2D ligands. Furthermore, activated NK cells had high level of PD-1 and could not kill PD-L1+ melanoma cells effectively. When we used PD-L1 inhibitor or silenced PD-L1 gene, inhibited PD-1/PD-L1 axis reversed the activity of the suppressed NK cells. Through these results, we supposed that PD-1/PD-L1 blockade could enhance the immune responses of NK cells against melanoma cells after radiotherapy and might overcome the PD-L1 mediated radioresistance of cancer cells.

## Introduction

Radiotherapy is a major modality in treatment of most common cancers including melanoma. Both pro-and anti-cancer immune responses could be induced in cancer microenvironment after radiation. The anti-cancer immune responses are observed in some cancers though upregulation of several immune stimulation genes such as TNF-α and release of antigenic proteins such as HSPs after radiotherapy in glioblastoma, breast cancer and melanoma [1–3]. However, it was known more recently that radiation promotes the remnant cancer cells to escape immune system and distant metastasis through the increased expression of TGF-β, PD-L1 and MMP-2 in cancer cells [4–6]. Furthermore, ionizing radiation may alter the anti-cancer activity of lymphocytes through dysregulation of immune check points molecules such as PD-1 and CTLA-4 [7, 8]. Therefore, these adverse effects of radiotherapy should be considered and managed to treat the cancer patients. Since it was known that radiotherapy could induce the PD-L1 in several cancer cells including head and neck squamous cell carcinoma, bladder cancer and non-small cell lung cancer [9–11], it was supposed that PD-1/PD-L1 axis blockade was required to inhibit the adverse effect of radiotherapy and may be benefit to treat cancer patients.

NK cells are critical innate immune lymphocytes to destroy virally infected or cancerous cells through targeted cytotoxicity [12]. Interestingly, we found that NK cells expressed PD-1 on cell surface and the level of PD-1 increased significantly during their activation. Therefore, it was supposed that NK cell-mediated immune responses were controlled by the negative signals through PD-1 as if the cancer reactive T cells did and its blockade might be required to obtain the sufficient anti-cancer immunity. In this study, we evaluated the efficacy on NK cell-mediated anticancer immune responses after irradiation and investigated the role of PD-1/PD-L1 axis in NK cells.

## Materials and methods

### Cell lines and reagents

Human melanoma cell line SK-MEL-28 was purchased from Korea Cell Line Bank (Seoul, Korea). Human melanoma cell line A375P and human chronic myelogenous leukemia cell line, K562, were purchased from the American Type Culture Collection (Rockville, MD, USA). A375P and SK-MEL-28 cell lines were maintained in DMEM media supplemented with 10% fetal bovine serum (FBS) (Gibco, Grand Island, NY), 2 mM L-glutamine, 100 mg/ml streptomycin, and 100 U/ml penicillin. The NK-92 cell line was purchased from American Type Culture Collection (Rockville, MD, USA) and maintained in α -Minimum Essential Modified medium supplemented with 12.5% (v/v) fetal bovine serum, 12.5% (v/v) horse serum, 2 mM L-glutamine, 0.1 mM 2-mercaptoethanol, 200 U/mL of recombinant human interleukin-2, 100 mg/mL streptomycin, and 100 U/mL penicillin. All cell lines were cultured at 37°C in a humidified atmosphere containing 5% CO2. Routine mycoplasma detection was performed in our laboratory and mycoplasma infection was not detected in regular quality test.

Experiments using human blood samples were approved by the Ethical Committee of Dongnam Institute of Radiological & Medical Sciences, and written informed consent was obtained from all the donors before enrollment (IRB No: D-2002-032-002). To obtain highly purified primary NK cells, non-NK cells were depleted by using EasySep^TM^ Direct Human NK Cell Isolation Kit (STEMCELL^TM^ Technologies, Vancouver, BC, Canada) according to the manufacturer’s instructions. Highly purified NK cells were expanded as previous study [13].

BMS202, PD-1/PD-L1 inhibitor 2 (Selleckchem, TX, USA), was dissolved in dimethyl sulfoxide (DMSO; Sigma-Aldrich, MO, USA) at 20 μM and used at 20 nM dose.

### Flow cytometry

Mouse anti-human CD273(PD-L2; #345505), CD274(PD-L1; #329709), CD279(PD-1; #329911) antibodies were purchased from BioLegend (San Diego, CA, USA). Cell-surface staining was performed by incubating tumor-derived cell populations with selected antibodies on ice in the dark for 30 minutes in PBS. To determine the surface PD-L1/2, PD-1 on cancer cells, the analysis was performed using BD FACS Canto™ II (Becton Dickinson, San Jose, CA USA). The data was analyzed by the Flowjo software (TreeStar, OR, USA) and the expression levels were determined by the Mean Fluorescence Intensity (MFI).

### Exposure to ionizing radiation

To irradiate the cancer cells, ClinaciX Linear Accelerator (Varian Medical Systems Inc., Palo Alto, CA, USA) was used under consults of Dr. Jiho Nam and Yong Gan Ki (Pusan National University Yangsan Hospital). The cells were irradiated with rate of 8 Gy/min under 10 mm depth-coverage of medium. Irradiated melanoma cells were recovered for 24 hours and then, the cells were collected to other assays.

### siRNA transfection

The siRNA used for the targeted silencing of PD-L1 and were purchased from (siNC; cat.no. 4390846; Thermo Fisher Scientific, Inc.). In brief, melanoma cells (2X10^5 cells/ml) were seeded on 6-well plates and transfected with 0.2 mM of siRNA using oligofectamine reagent, according to the manufacturer’s instructions (Invitrogen, Carlsbad, CA, USA). Cells were then incubated at 37°C for 4 hours in serum free media and FBS was added to 10%. After 48 hours, cells were collected for Cytotoxicity assay.

### NK cell-mediated cytotoxicity

NK cell-mediated cytotoxicity was determined using FC500 flow cytometer (Beckman Coulter, CA, USA). The melanoma cells were stained with 50 mM carboxyfluorescein succinimidyl ester (CFSE) for 30 min at 37˚C and washed three times. NK-92 cells and CFSE-stained melanoma cells were co-cultured for 4 hours. Propidium iodide (PI) was added to the co-cultured samples for identification of the dead cells. Cytotoxicity were calculated by formula of (CFSE^+^PI^+^ cells / CFSE^+^ cells) X 100 (%).

### Statistical analysis

To evaluate the altered gene expression levels, the mean folds of gene expressions among the groups and the standard error of the mean were calculated. For comparisons of the groups, a paired Student’s t test was performed. The analysis was performed using the SPSS statistical package (version 14.0; SPSS Inc., IL, USA). P<0.05 indicates a statistically significant difference in all of the experiments.

## Results

### Surface PD-L1 and PD-L2 were increased by ionizing radiation with dose-dependent manner in melanoma cells

**Fig 1:**
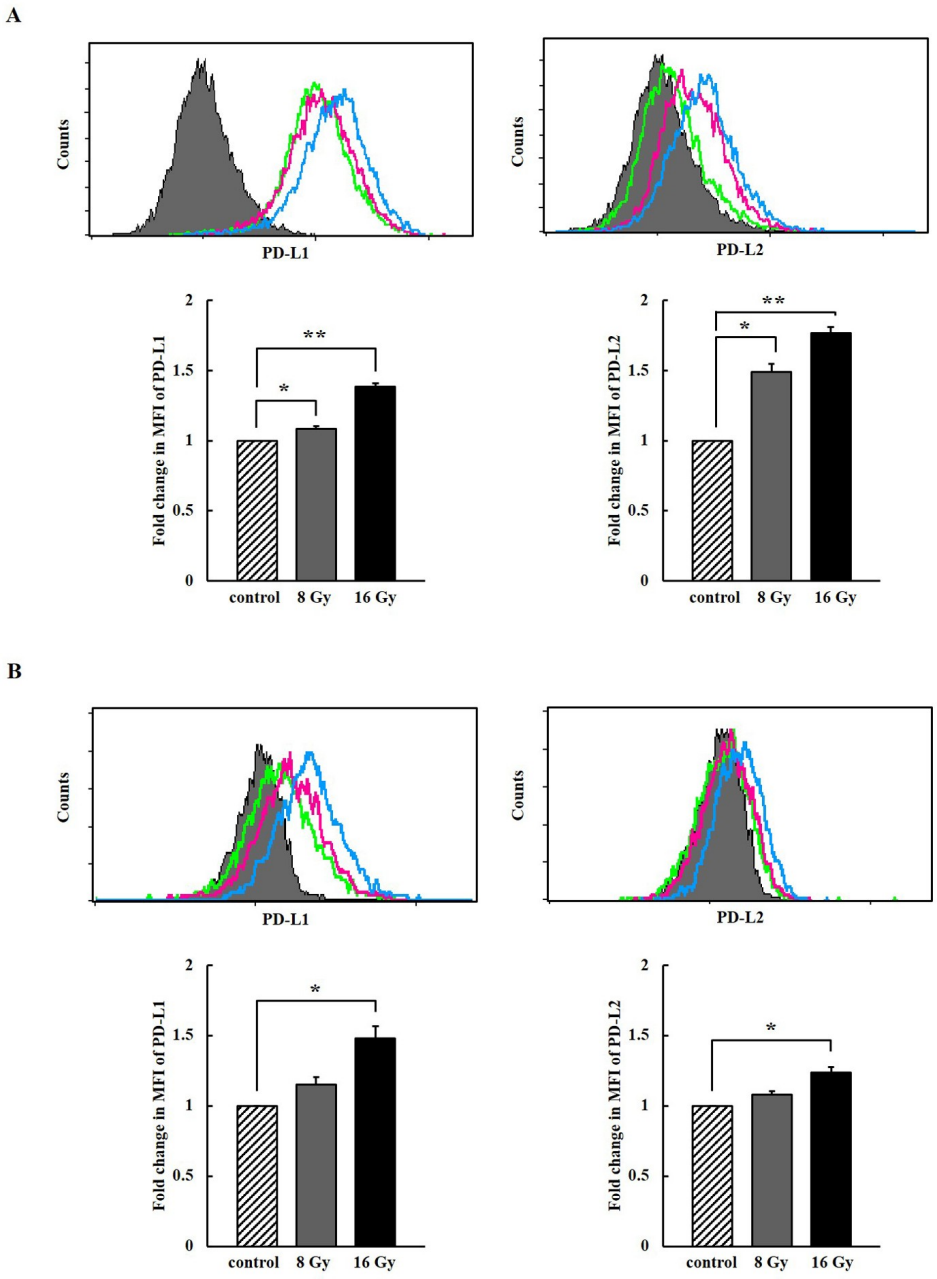
Induced surface expression of programmed death ligand 1/2(PD-L1/2) in (A)A375P and (B)SK-MEL-28 melanoma cells by ionizing radiation. The upper panels show a representative flow cytometry data (filled gray–isotype; green line–control; purple line– 8 Gy; blue line– 16 Gy). The lower panels show fold changes in Mean Fluorescence Intensities (MFIs) (diagonal line–control; filled gray– 8Gy; filled black– 16 Gy). The experiments were performed three times. (*p* < 0.05, *; *p* < 0.01, **; *p* < 0.001, ***).

The surface expression of PD-L1/2 in melanoma cells was detected using PE-conjugated anti-PD-L1/2 antibodies and analyzed by flow cytometry. The expression level of PD-L1 was generally high and PD-L2 was detected at low level in A375 cells and SK-MEL-28 cells. The expression of PD-L1 and PD-L2 were increased following 8 Gy and 16 Gy irradiation in a dose-dependent manner in two melanoma cells (Fig 1). It was suggested that ionizing radiation may be a potent inducer of two ligands of PD-1 and high dose irradiation may have adverse effects in anti-cancer immunity.

### The expression NKG2D ligands increased by ionizing radiation in melanoma cells

**Fig 2:**
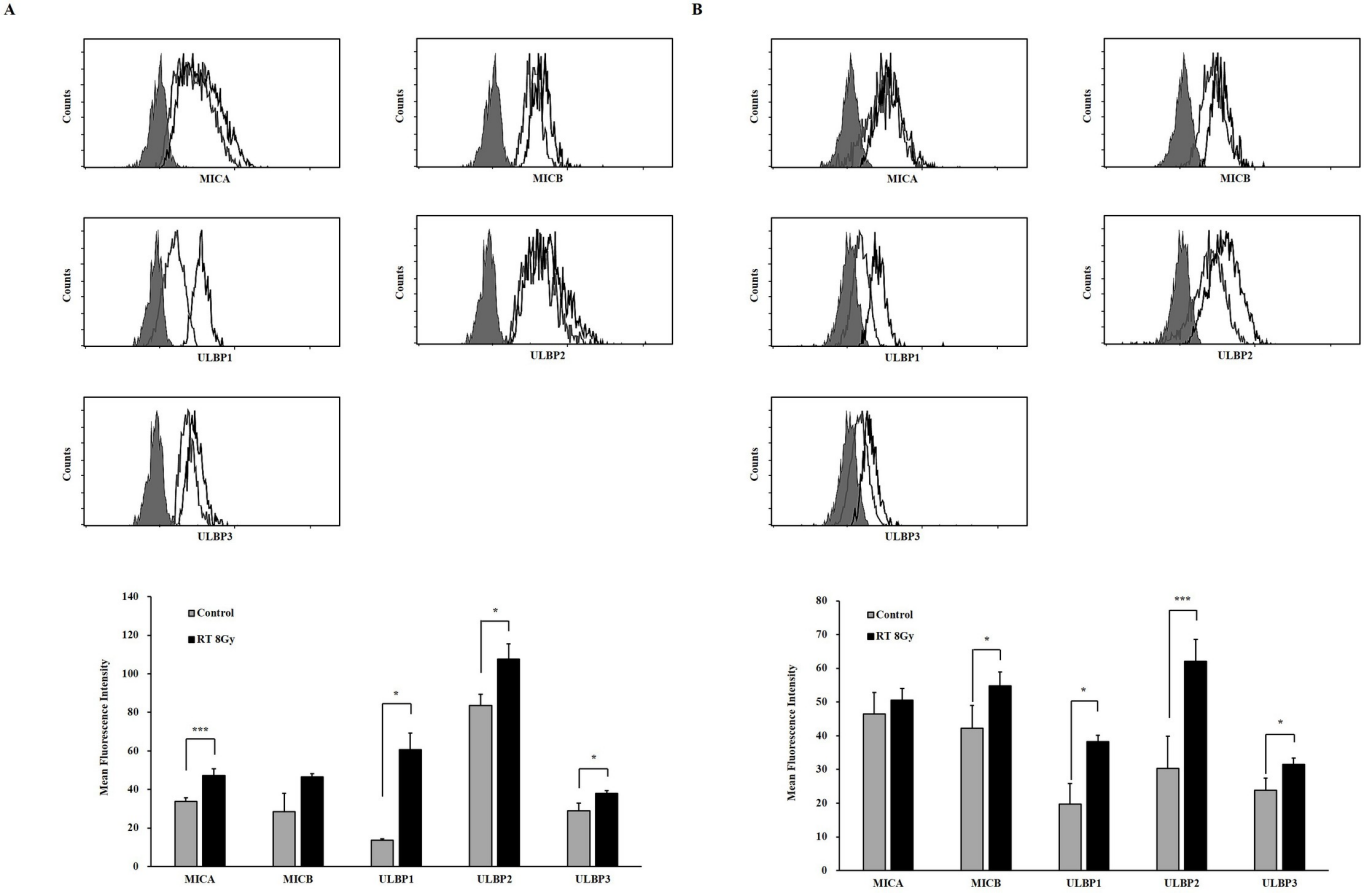
Induced surface expression of NKG2D ligands in (A) A375P and (B) SK-MEL-28 melanoma cells by ionizing radiation. The upper panels show a representative flow cytometry data (filled gray–isotype; gray line–control; black line– 8Gy) and the lower panels show MFIs (gray–control; black– 8Gy). The experiments were performed three times. (*p* < 0.05, *; *p* < 0.001, ***).

It was well known that the DNA damage agents including ionizing radiation could increase the NKG2D ligands by facilitation protein translation through ATM-ATR pathway [14]. To determine these changes on melanoma cells, the surface expression of NKG2D ligands including MICA, MICB and ULBP1-3 were detected using mouse anti-human specific antibodies and goat PE-conjugated anti-mouse IgG secondary antibodies. The expression of five NKG2D ligands were increased following 8 Gy irradiation in two melanoma cells (Fig 2). We confirmed the same radiation effects on melanoma cells. Contrary to PD-1, these NKG2D ligands could engage the activity of NKG2D+ immune cells.

### The expression of PD-1 increased dramatically during activation of NK-92 cells

**Fig 3:**
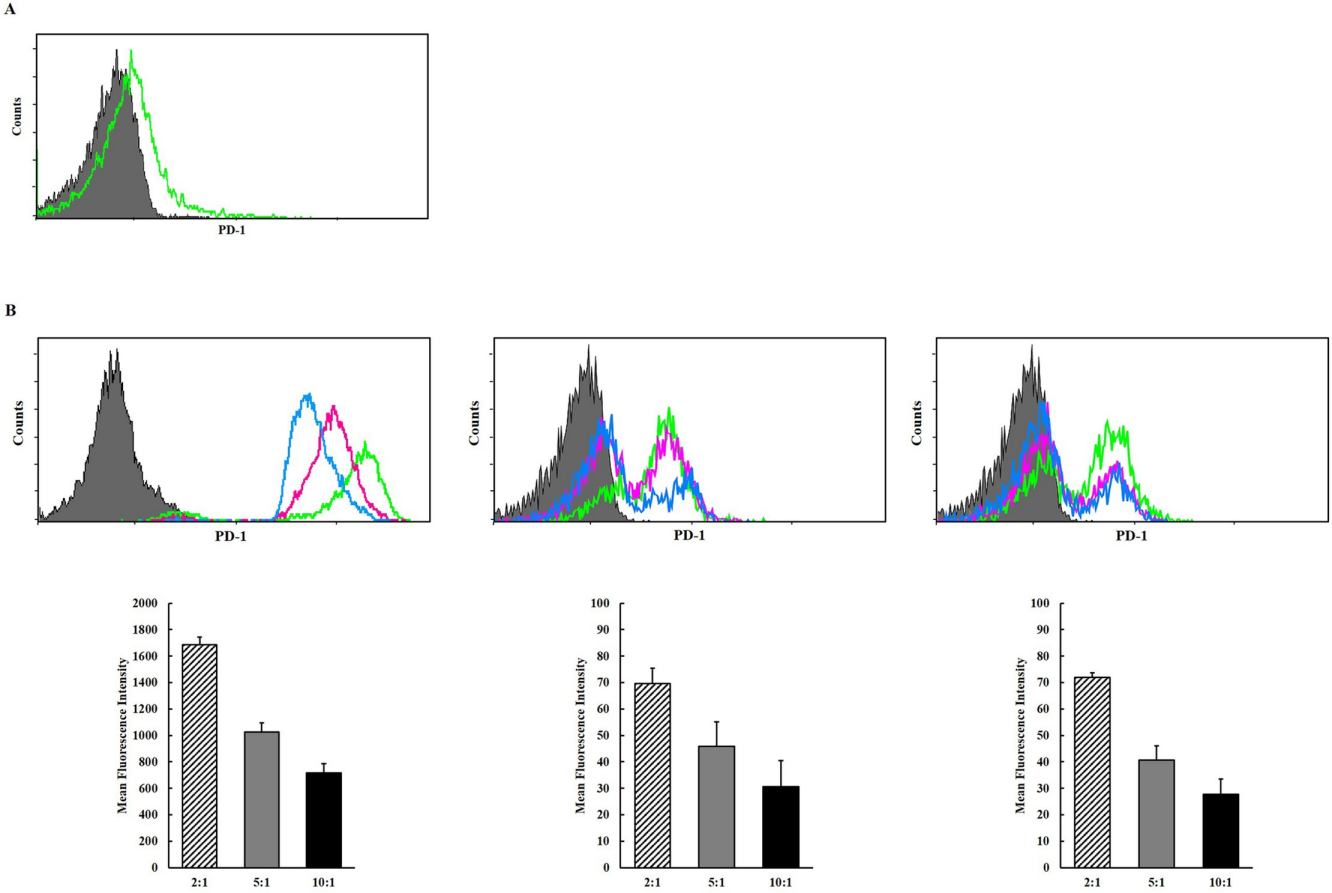
(A) Surface expression levels of programmed death 1 (PD-1) in steady state NK cells. (B) Surface expression of PD-1 in activated NK-92 cells following co-culture with K562 cells and melanoma cells (A375P, SK-MEL-28), respectively, at each E:T ratio. The upper panel shows representative histograms (green line– 2:1; purple line– 5:1; blue line– 10:1) and the lower panel shows MFIs (diagonal line– 2:1; filled gray– 5:1; filled black– 10:1). The experiments were performed three times.

Although NK-92 cells in steady state expressed a little amount of PD-1 (Fig 3A), the expression of PD-1 was dramatically increased when they are stimulated by K562 cells and melanoma cells (Fig 3B and 3C). It seems that active NK cells might be intrinsically suppressed to prevent uncontrolled immune responses by induction of PD-1 and it makes be hard to maintain the potent cytotoxic activity for sufficient time against cancer cells physiologically. Therefore, it was supposed that PD-L1^high^ cancer cells might survive through escape from NK cell-mediated immune responses.

### The susceptibility of melanoma cells to NK cells is increased by PD-L1 inhibitor

**Fig 4:**
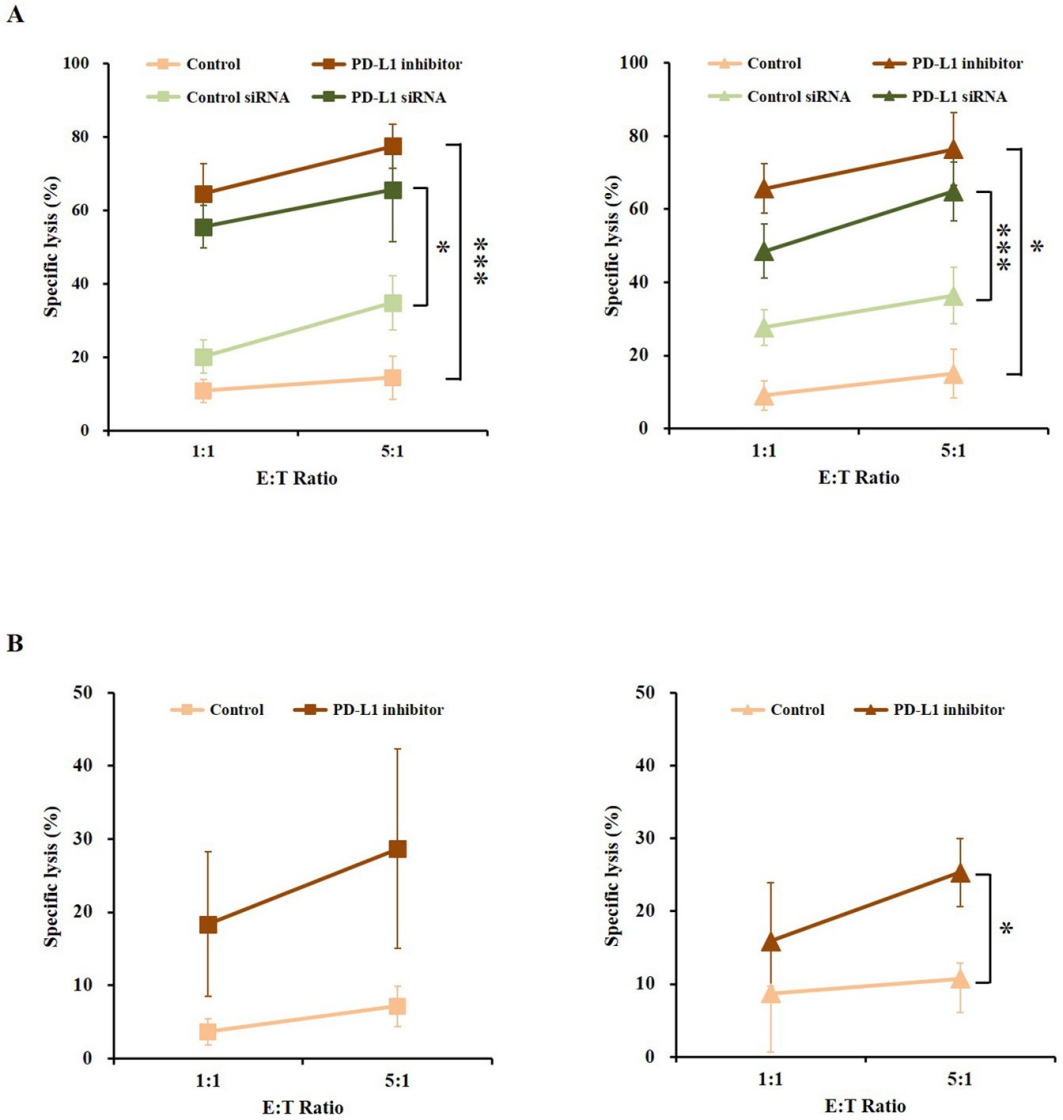
Enhanced susceptibility of A375P (left panels) and SK-MEL-28 (right panels) human melanoma cancer cells to (A) NK-92 and (B) primary NK cells by treatment with PD-L1 inhibitor or siRNA PD-L1 (light brown–control; light green–control siRNA; dark green–PD-L1 siRNA; dark brown–PD-L1 inhibitor, respectively.). The experiments were performed three times. (*p* < 0.05, *; *p* < 0.001, ***).

The high expression of PD-L1 in melanoma cells and increased PD-1 in NK cells might repress the activity of NK cells after short period of action and it seems that NK cells did not have enough activity to eliminate melanoma cells. By using silencing of PD-L1 gene or treatment of PD-L1 inhibitor, we supposed that anti-cancer immune responses of NK cells against melanoma cells could be altered. The susceptibility of melanoma cells to NK-92 cells was significantly enhanced by inhibition of PD-1/PD-L1 axis (Fig 4A). Furthermore, the susceptibility of melanoma cells to primary NK cells was enhanced by PD-L1 inhibitor (Fig 4B).

## Discussion

Anti-PD-1 agents have been the central molecule for cancer treatment such as melanoma and have shown synergy with radiotherapy [2]. Administration of anti–PD-L1 enhanced the efficacy of IR through a cytotoxic T cell–dependent mechanism and showed cancer regression. It was reported that IR and anti–PD-L1 synergistically reduced the local accumulation of tumor-infiltrating myeloid-derived suppressor cells (MDSCs), which suppress T cells and alter the tumor immune microenvironment mouse colon cancer [3]. The combination of PD-1 blockade and localized radiation therapy results in long-term survival in mice with orthotopic brain tumors mouse glioma [1]. The immune checkpoint PD-1 is expressed on many cancer infiltrating lymphocytes in response to inflammation. The engagement of PD-1 on the lymphocyte by PD-L1 on melanoma cells downregulates T-cell function and might promote exhaustion of cancer reactive T cells [15, 16]. The usage of anti–PD-1 and anti–PD-L1 antibodies has been remarkably successful, both in terms of response rates (30%–45%) and durability (2–3 years) in melanoma [2, 17–24], even after discontinuation of the treatments [2, 25]. These enthusiastic studies about PD-1/PD-L1 have been focused on adaptive immune cells, especially T cells. The role and regulation of PD-1/PD-L1 in innate immunity remains poorly understood until recent. Recently, the role and regulation of PD-1/PD-L1 in innate immunity have been reported little by little [12, 16]. Furthermore, the emerging data indicate that combining PD-L1 inhibitors with other therapies including chemotherapy, radiotherapy and immunotherapy might be more beneficial to cure cancer patients [26–28] and we already have been studied that ionizing radiation was efficient modality to trigger NK cell-mediated immune responses [29]. Therefore, we evaluated the efficacy on NK cell-mediated anticancer immune responses by the combination with PD-L1 inhibitor. Activation of NK cells rapidly induced PD-1 receptor on their surface and irradiated melanoma cells also expressed high level of ligands of PD-1. Therefore, it was supposed that the blockade of PD-1/PD-L1 was required to maintain the NK cell-mediated anti-cancer immunity against melanoma cells. We used the ionizing radiation to trigger the immune responses against melanoma cells by induction of NKG2D ligands, a kind of NK cell activating molecules. It was known that radiotherapy could induce the PD-L1 in several cancer cells including HNSCC, bladder cancer and NSCLC [9–11]. However, the mechanisms to induce the PD-L1 is not understood yet. It required more sophisticated strategies to find clues to resolve it. Considering these facts, we hypothesized that blocking of PD-L1 could enhance natural killer cell-mediated anticancer immunity to melanoma cell lines.

## Supporting information

S1 Fig (XLSX)

S2 Fig (XLSX)

S3 Fig (XLSX)

S4 Fig (XLSX)
